# Complete genome sequence of *Sedimentibacter* sp. strain MB31-C6, isolated from sewage sludge

**DOI:** 10.1128/mra.00077-26

**Published:** 2026-03-16

**Authors:** Hangying Zhang, Juncheng Zhang, Yin Li, Li Yang, Chao-Jen Shih, Yen-Chi Wu, Sheng-Chung Chen

**Affiliations:** 1 Fujian Provincial Key Laboratory of Resources and Environmental Monitoring and Sustainable Management and Utilization, Sanming University 66283, Sanming, Fujian, People’s Republic of China; 2 Medical Plant Exploitation and Utilization Engineering Research Center, Sanming University 66283, Sanming, Fujian, People’s Republic of China; 3 School of Resources and Chemical Engineering, Sanming University 66283, Sanming, Fujian, People’s Republic of China; 4 Bioresource Collection and Research Center, Food Industry Research and Development Institute, Hsinchu, Taiwan, Republic of China; Nanchang University, Nanchang, Jiangxi, China

**Keywords:** *Sedimentibacter*, sewage sludge

## Abstract

Here, we report the complete genome of *Sedimentibacter* strain MB31-C6 (=BCRC 81431), isolated from sewage sludge at the Sanming Steel wastewater treatment plant, China. The strain harbors a 3,009,901 bp circular chromosome (30.1% GC) and a 13,652 bp plasmid, which were used for further species delineation and comparative genomic analyses.

## ANNOUNCEMENT

**Fig 1:**
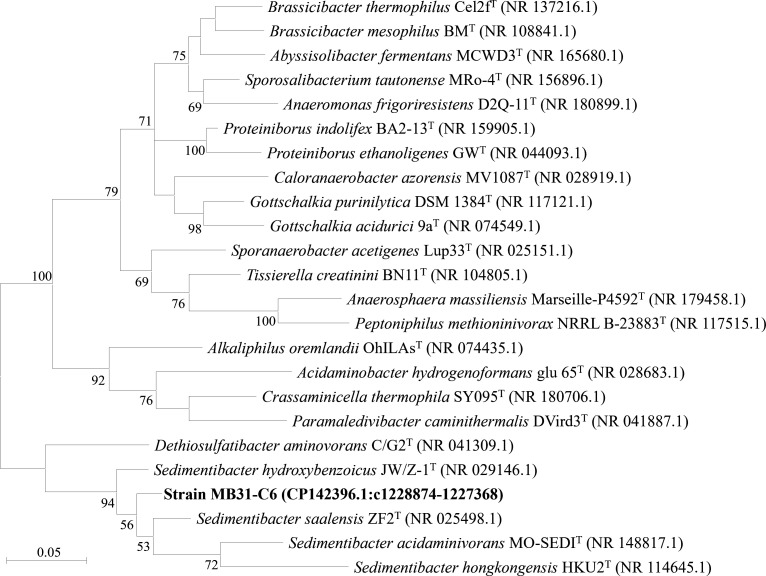
Maximum Likelihood tree based on 16S rRNA gene sequences of strain MB31-C6 and related taxa. Sequence alignment was performed using Clustal W (9), and the Tamura-Nei model (10) was applied as the nucleotide substitution model. Bar, 0.05 substitutions per nucleotide position. Bootstrap values were expressed as percentages of 1,000 replicates.

Species of the genus *Sedimentibacter* are obligately anaerobic, spore-forming bacteria commonly found in organic-rich anoxic habitats, where they participate in amino acid fermentation and carbon and nitrogen cycling (1–4). Their recovery from both environmental samples and human blood suggests a broad ecological distribution and potential functional versatility (1–4). In this study, the strain MB31-C6 was isolated from dried sewage sludge of the Wastewater Treatment Plant of Sanming Steel Co. Ltd, Fujian, China. Sewage sludge was collected on 25 June 2021. The sample of sludge was inoculated into the anaerobic modified DSM 924 medium (per liter: 1 g MgCl_2_·7H_2_O, 0.5 g KCl, 0.1 g CaCl_2_·2H_2_O, 0.4 g K_2_HPO_4_, 1 g NH_4_Cl, 10 mL trace element solution, 2 g yeast extract, 2 g tryptone, 0.5 mL Na-resazurin solution [0.1%], 4 g NaHCO_3_, 10 mL vitamin solution, 0.25 g L-cysteine-HCl·H_2_O, 0.25 g Na_2_S·9H_2_O, and headspace: N_2_:CO_2_ = 4:1), prepared according to the instruction of medium and incubated at room temperature (~25℃) for 2 weeks. To further purify and identify the strain MB31-C6, a combined approach was employed, integrating serial dilution, rolling-tube technique (5), single-colony isolation, and colony PCR-based 16S rRNA gene clone sequencing using 8F and 1492RU primers (6). Bacterial purity was confirmed through morphological observation, 16S rRNA gene sequencing, and genome sequencing. Based on the 16S rRNA gene analysis through BLASTN (7), strain MB31-C6 showed highest similarities to more closely related valid strains, *Sedimentibacter acidaminivorans* MO-SEDI^T^ (NR_148816, 95.29% identity; query coverage, 94%) (1), *Sedimentibacter saalensis* ZF2^T^ (NR_025498, 95.00% identity; query coverage, 95%) (2), *Sedimentibacter hydroxybenzoicus* JW/Z-1^T^ (NR_029146, 94.71% identity; query coverage, 96%) (3), and *Sedimentibacter hongkongensis* HKU2^T^ (NR_114645.1, 92.19% identity; query coverage, 94%) (4). Phylogenetic analysis of 16S rRNA gene sequences performed by MEGAX (8) for strain MB31-C6 and related taxa indicated that strain MB31-C6 could be a novel lineage (Fig. 1).

The genomic DNA of strain MB31-C6 was extracted using the QIAGEN Genomic-tip 20/G kit according to the manufacturer’s instructions. The DNA was quantified and qualified using a NanoDrop 2000 spectrophotometer and Qubit TM3 fluorometer (Thermo Fisher Scientific). Library preparation involved shearing 15 µg of DNA with g-TUBE (Covaris) according to the manufacturer’s protocol to achieve a target fragment size of ~15 kb, as required for PacBio long-read sequencing. Small fragments (<1 kb) were removed using AMPure PB Beads to enrich larger fragments and improve library quality. The SMRTbell library was constructed following the instructions of the SMRTbell Express Template Preparation Kit 2.0 (PacBio, Cat No. 100-938-900) and sequenced at the Biomarker Technologies (BMKGENE) Co., Ltd. (Beijing) using the PacBio Sequel II sequencer (v2.0 chemistry, Pacific Biosciences).

Raw subreads obtained from the PacBio Sequel II platform were processed using the SMRT Link v10.1 (Pacific Biosciences) to generate circular consensus sequences (CCS). Low-quality and short reads (<2,000 bp) were filtered out, and the resulting 48,609 high-quality reads (total base, 336,259,232 bp; N_50_, 7,684 bp) were used for subsequent *de novo* assembly. Genome assembly was carried out using Hifiasm v0.16.1 (11). Circularization of the genome was verified using Circlator v1.5.5 (12), which identified overlapping ends and confirmed complete circular topology. Contigs shorter than 1 Mb were preliminarily regarded as plasmids. The result yielded two contigs, including a 3,009,901 bp chromosome (30.1% GC; sequencing coverage, 111.7×) and a 13,652 bp plasmid (25.7% GC; sequencing coverage, 24,630.8×). The NCBI Prokaryotic Genome Annotation Pipeline (PGAP, v6.7) was used for functional annotation (13). The chromosome was predicted to have 2,919 genes, of which 2,819 were protein coding, and contains 15 rRNA genes and 63 tRNA genes. In addition, strain MB31-C6 exhibited 75.19% average nucleotide identity (ANI) with its closest validly published species, *S. acidaminivorans* MO-SEDI^T^, calculated using the EZBioCloud ANI Calculator (14), consistent with the 16S rRNA gene-based analysis. Default parameters were applied in all bioinformatics analyses.

## Data Availability

The sequences of genome and plasmid of strain MB31-C6 have been deposited in GenBank under accession number CP142396.1 and CP142397.1, respectively. The version of the assemblies described in this paper is the first version. The BioProject and BioSample accession numbers are PRJNA1054745 and SAMN38931393, respectively. The raw sequence reads were deposited in the Sequence Read Archive (SRA) under accession number SRR36877252.
